# *Bacteroides fragilis* 839 ameliorates anti-tuberculosis drugs-induced liver injury by suppressing inflammation and regulating gut microbiota in mice

**DOI:** 10.3389/fmed.2025.1538528

**Published:** 2025-04-17

**Authors:** Qiujuan Li, Chenbing Wu, Kangshuai Zhang, Ziyi Zhou, Jing Li, Jie Bai, Jun Cao, Xiaoxia Shi

**Affiliations:** ^1^Department of Experimental Teaching Center of Public Health, Dalian Medical University, Dalian, China; ^2^Department of Pathology and Forensic Medicine, Dalian Medical University, Dalian, China; ^3^Department of Occupational and Environmental Health, Dalian Medical University, Dalian, China

**Keywords:** *Bacteroides fragilis* 839, anti-tuberculosis drugs, liver injury, intestinal barrier, LPS/TLRs/NF-κB pathway

## Abstract

Anti-tuberculosis drug-induced liver injury (ATB-DILI), caused by first-line anti-tuberculosis (anti-TB) drugs, disrupts treatment and increases the risk of drug resistance. The gut microbiota and intestinal barrier integrity play key roles in ATB-DILI susceptibility through the liver-gut axis. Probiotics, such as *Bacteroides fragilis* 839 (BF839), have shown therapeutic potential in modulating gut microbiota and inflammatory responses. In this study, we investigated the protective effects of BF839 on ATB-DILI in a mouse model of HRZE-induced liver injury. BF839 administration significantly alleviated HRZE-induced liver injury by reducing ALT, AST, AKP, and MDA levels, enhancing SOD and GSH levels, and improving liver histopathology. These effects were associated with restored gut microbiota diversity, enhanced intestinal barrier function, and reduced inflammatory responses. Our findings suggest that BF839 may serve as a potential preventive strategy for ATB-DILI.

## Graphical abstract

*Bacteroides fragilis* 839 (BF839) could protect against liver injury induced by isoniazid, rifampicin, pyrazinamide and ethambutol (HRZE) by recovering gut microbiota balance, improving intestinal barrier function, and suppressing inflammatory response through the regulation of the lipopolysaccharid (LPS)/Toll-like receptor 4 (TLR4)/NF-κB signaling pathway.

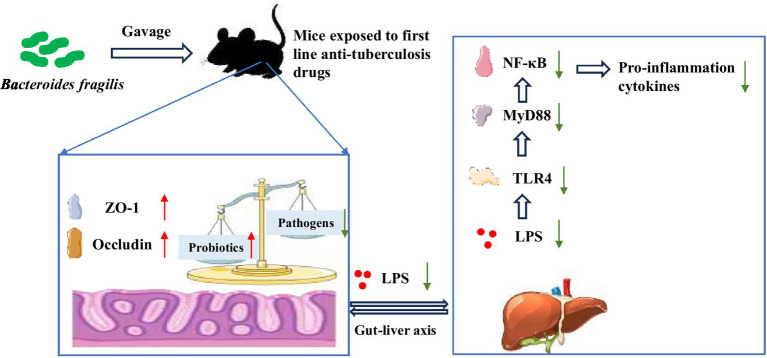

## Highlights

- *Bacteroides fragilis* 839 (BF839) effectively alleviates ATB-DILI in mice.
- BF839 restored the balance of the gut microbiota and repaired the intestinal barrier.
- BF839 suppressed the inflammatory response by down-regulating LPS/TLR4/NF-κB pathway.

## 1 Introduction

Tuberculosis (TB), as an infectious disease caused by *Mycobacterium tuberculosis*, remains a major public health issue, posing a significant threat to human health (1). Currently, most TB patients respond well to standard HRZE regimens, which include a two-month course of isoniazid, rifampicin, pyrazinamide, and ethambutol, followed by a four-month course of rifampicin and isoniazid (2). However, prolonged antibiotic treatment often results in various adverse drug reactions, with anti-tuberculosis drug-induced liver injury (ATB-DILI) being the most common and severe. Studies indicate that that most ATB-DILI patients experience elevated liver enzymes, with severe cases leading to ascites and coagulation dysfunction (3). The incidence of ATB-DILI in patients during anti-tuberculosis (anti-TB) therapy ranges from 2.0 to 28.0%, with higher rates in China (9.5–14.4%) (4, 5). ATB-DILI is a key factor contributing to treatment interruptions or failures and the development of drug resistance.

The intestinal microbiota consists of over 1,000 species of bacteria, fungi, archaea, parasites, and viruses, with bacteria being the predominant component (6). The core bacterial phyla in human intestinal microbiota are Firmicutes, Bacteroidetes, Proteobacteria, and Actinomycetes, which together comprise up to 100 billion microorganisms. The intestinal microbiota exists in symbiosis with its host, and changes in its composition reflect the progression of the host’s disease. Recent studies have highlighted associations between gut microbiota and drug-induced liver injury (DILI), including those induced by 4-acetaminophen and tacrine (7, 8). Additionally, anti-TB drug therapy may trigger intestinal dysbiosis, impair gut barrier function, and promote the migration of harmful bacteria to the liver, thereby disrupting liver immune homeostasis and exacerbating liver damage. Namasivayam et al. reported that while mice infected with *M. tuberculosis* showed only slight alterations in their gut microbiota (9), liver damage caused by anti-TB treatment led to significant changes, including a decrease in beneficial bacteria and an increase in selective infections (10). Intestinal barrier markers, such as FITC-dextran and the tight junction protein Occludin, were reduced after ATB-DILI, whereas endotoxin levels and inflammatory markers sharply elevated (11). These findings suggest a bidirectional relationship between DILI and intestinal dysbiosis, where an imbalanced microbiota contributing to DILI progression. Therefore, restoring the diversity of the gut microbiota may present a novel therapeutic strategy for DILI.

Probiotics are considered to effectively modulate gastrointestinal function, restore the intestinal mucosal barrier, regulate immune function, and inhibit the growth of harmful bacteria. Studies showed that probiotics offer beneficial effects in DILI. For example, in animal models, *Lactobacillus rhamnosus* has been shown to activate Nrf2, an antioxidant factor, reducing liver damage caused by ethanol and acetaminophen (12). Akkermansia and its extracellular vesicles have also been found to reduced liver damage induced by CCL4 and ibuprofen by regulating of intestinal permeability and gut immunity (13, 14). Therefore, probiotics are a potential adjuvant treatment for DILI. However, there are few studies examining their effects on ATB-DILI.

*Bacteroides fragilis* (*B. fragilis*), an essential obligate anaerobe in the lower gastrointestinal tract and exists in two subtypes: enterotoxigenic and non-enterotoxigenic. The enterotoxigenic subtype is linked to diarrheal disease (15, 16). Non-enterotoxigenic strains, such as *B. fragilis* 839 (BF839), are considered potential probiotics that may reduce colonic inflammation. BF839 has demonstrated efficacy in preventing gastrointestinal side effects and myelosuppression caused by chemotherapy in cancer patients (17, 18). BF839 has also been shown to exert anti-inflammatory effects by modulating cytokine expression and gut microbiota balance in psoriasis patients (17, 18). Recent research suggests that BF839 may also alleviate gastrointestinal issues related to autism spectrum disorder (19). Therefore, we hypothesize that this strain BF839 may improve gastrointestinal symptoms related to ATB-DILI and correct the gut microbiota imbalance, making it a promising candidate for the prevention and/or treatment of ATB-DILI. In this study, we established a mouse model to investigate the potential of BF839 as a treatment. Liver damage and gastrointestinal barrier function were assessed by western blotting and hematoxylin and eosin (H&E) staining, while the composition and diversity of gut microbiota were analyzed by 16S rRNA gene sequencing. The findings of this study may demonstrate the potential of BF839 as a probiotic for DILI patients.

## 2 Methods and materials

### 2.1 Animal treatment

In this study, BF839 was provided by them for free. Dalian Totem Life Science Development Co., Ltd.

A total of thirty C57BL/6J male mice (8 week old) were purchased from Liaoning Changsheng biotechnology. The mice had free access to water and were fed a diet outlined in Table 1. The mice were housed in a specific pathogen-free (SPF) barrier facility at a controlled temperature of 22–26°C, with a daily temperature variation of no more than 4°C. During the experiment, the relative humidity was maintained at 40–70%, and the air cleanliness level was kept at grade seven. Noise levels were controlled below 60 dB, and a 12-h light/dark cycle was implemented. All animal experiments were conducted in accordance with the NIH guidelines and were approved by Dalian Medical University’s Ethics Committee for Animal Experiments (permit number: AEE23078).

**Table 1:** Formulation of standard diet.

Ingredient	Mass (g/kg)
Casein	200
L-cystine	3
Sucrose	100
Cornstarch	397.486
Dyetrose	132
Soybean oil	70
Cellulose	50
Mineral mix	35
Vitamin mix	23.2
Total	1000.0

Before the formal experiment began, the mice were adaptively fed for 1 week. Then, the mice were randomly divided into three groups: the control group, the HRZE group, and the HRZE + BF839 group. Mice in the control group were administered 0.5% sodium carboxymethyl cellulose, and then 0.9% normal saline 4 h later. Mice in the HRZE group received a mixture of HRZE, containing 0.15 g/kg isoniazid, 0.3 g/kg rifampicin, 0.63 g/kg pyrazinamide and 0.38 g/kg ethambutol (dissolved in 0.5% sodium carboxymethyl cellulose solution), followed by 0.9% normal saline 4 h later. Mice in the HRZE + BF839 group were administered HRZE mixture, then, given a gavage of BF839 (>10^9^ CFU/day/mouse) dissolved in distilled water. All animals were treated by intragastric administration for six consecutive weeks. Animals were sacrificed the morning following the last administration. Serum, liver, colon tissues and fecal samples were collected and stored at −80°C for further analysis.

### 2.2 Aspartate aminotransferase (AST), alanine aminotransferase (ALT), alkaline phosphatase (AKP) assays

Serum samples were used to detect the following indicators: The levels of ALT and AST were measured by the ALT Assay Kit (Nanjing Jiancheng Bioengineering Institute, Cat: C009-2-1) and the AST Assay Kit (Nanjing Jiancheng Bioengineering Institute, Cat: C010-2-1). The level of AKP was measured using the AKP Assay Kit (Nanjing Jiancheng Bioengineering Institute, Cat: A059-2).

### 2.3 Superoxide dismutase (SOD), malondialdehyde (MDA), glutathione (GSH) content detection

The total protein of mouse liver samples was extracted using phosphate buffer solution PBS. The protein concentrations of the samples were measured prior to further analysis. The levels of SOD, MDA, and GSH were quantified using commercially available kits (Beyotime Biotechnology, Nantong, China, Cat: S0101S; Cat: S0131S; Cat: S0053). All experimental procedures strictly followed the manufacturer’s instructions.

### 2.4 H&E staining

After the C57BL/6J male mice were sacrificed, the liver and intestinal tissues were immediately fixed in 10% neutral-buffered formalin. The tissues were then embedded in paraffin and sectioned into thin slices. Paraffin sections with a thickness of 4 μm were dewaxed, rehydrated, and stained with H&E. The stained sections were observed and analyzed under a light microscope, following proper adjustment of the magnification.

### 2.5 Enzyme-linked immunosorbent assay (ELISA)

Total protein of mice livers was extracted by phosphate buffer solution which added 1 mM phenylmethanesulfonyl fluoride (PMSF). The protein concentration of the samples was measured using the BCA Protein Assay Kit. Liver levels of the tumor necrosis factor-alpha (TNF-α), interleukin (IL)-1b and, IL-6 were quantified by ELISA kits (MULTI SCIENCES, Cat: EK282/4–96; Cat: EK201B/3–96; Cat: EK206/3–96). Additionally, serum lipopolysaccharide (LPS) levels were measured by the LPS Assay Kit (Ruixin Biotech, Cat: RX202425M).

### 2.6 Western blotting analysis

Total protein of mice livers and intestines was extracted using RIPA (Solarbio, R0020) containing 1 mM PMSF. Protein concentration was determined using the BCA Protein Quantification Kit (Nanjing Vazyme Biotech Co., Ltd., Cat: MK164230). The samples were separated by sodium dodecyl sulfate-polyacrylamide gel electrophoresis (SDS-PAGE), and were transferred onto 0.45 μm polyvinylidene fluoride (PVDF) membranes (Millipore, IPVH00010). The membranes blocked with 10% nonfat milk at room temperature for 2 h. Tris-buffered saline Tween-20 (TBST) was used to dilute the antibodies to the recommended concentrations. After blocking, the membranes were incubated in primary antibodies at 4°C for 12 h, and then incubated with secondary antibodies for an hour at room temperature. The following antibodies were used in the experiment: β-Actin (1: 5000, Proteintech, Cat: 66009-1-Ig); Toll-like receptor 4 (TLR4) (1:1000, Proteintech, Cat:66350-1-Ig); *myeloid differentiation factor* 88 (MyD88) (1:500, Wanleibio, WL02494); nuclear factor kappa B (NF-κB) (1:5000, Cat: Abmart, T55034); phosphorylated NF-κB (p- NF-κB) (ser536) (1:1000, Bioss, Cat: bs17502R); zona occludens (ZO-1) (1:500, Cat: Wanleibio, WL03419); Occludin (1:1000, Cat: Wanleibio, WL01996); Goat Anti-rabbit IgG (1:5000, Cat: Bioss, bs-0295G-HRP).

To ensure accurate detection, strip analysis was performed on the same membrane in two stages. The membrane was first probed with primary and secondary antibodies for the low-abundance target protein. Afterward, antibodies were removed using a stripping buffer (Cat: SW3020, Solarbio). After stripping, the membrane was re-incubated with a new primary antibody to detect the internal control protein (β-Actin).

Due to the significant difference in molecular weight between ZO-1 (approximately 220 kDa) and β-actin (approximately 42 kDa), which require different transfer times, the gels were divided into two parts: one for ZO-1 and the other for β-actin. Both ZO-1 and β-actin were then analyzed on the same membrane, following the same procedure described earlier.

The protein bands were detected using the BioRad ChemiDoc™ MP imaging system. Band intensity was quantified using ImageJ software (NIH). The expression of the target proteins was represented as the ratio of gray values between the target protein and β-actin, which served as the loading control.

### 2.7 16S rRNA sequencing

Fecal samples were obtained at weeks two and six. DNA was extracted and purified from these samples, and the 16S rRNA V3-V4 region was amplified using universal primers (16S V3-V4: CCTAYGGGRBGCASCAG, GGACTACNNGGGTATCTAAT). After purification, amplicons were used to create sequencing libraries and were sequenced on the Illumina NovaSeq6000 PE250 platform (Beijing Novogene Co., Ltd).^1^ Paired-end reads were generated and filtered using FLASH (V1.2. 11) and fastp (Version 0.23.1) software. The reads were then merged, denoised, and clustered into high-confidence Amplicon Sequence Variants (ASVs) using the DADA2 plugin in the QIIME2 software (Version QIIME2-202202). Taxonomic assignment was performed using the SILVA reference database. Subsequent diversity analyses, including alpha and beta diversity, were based on the ASV table. To analyze the diversity, richness and uniformity of the communities of every group, alpha diversity was assessed using the Shannon and Chao1 indices in QIIME2. To evaluate the complexity of the community composition and compare the differences between groups, beta diversity was visualized using PCoA analysis based on unweighted unifrac distances in QIIME2. Statistical comparisons of alpha diversity were performed using one-way ANOVA, and beta diversity differences were assessed using PERMANOVA. Additionally, the analysis of multi-omics correlation was performed using BMKCloud.^2^

### 2.8 Statistical analysis

The data were expressed as the means ± standard error of the mean (SEM). To ensure the reliability of these experiments, the experiments were repeated at least three times. Statistical analyses were performed by SPSS 26.0 software. Data were analyzed using one-way analysis of variance (ANOVA) to compare differences between multiple groups. For gut microbiota data, alpha diversity was assessed using the Chao1and Shannon indices, with statistical differences evaluated by one-way ANOVA, followed by Tukey’s *post hoc* test for pairwise comparisons. Beta diversity was assessed using unweighted uniFrac distances, with statistical significance determined by PERMANOVA. A *p* < 0.05 was considered statistically significant.

## 3 Results

### 3.1 BF839 alleviated liver injury induced by HRZE

**Figure 1:**
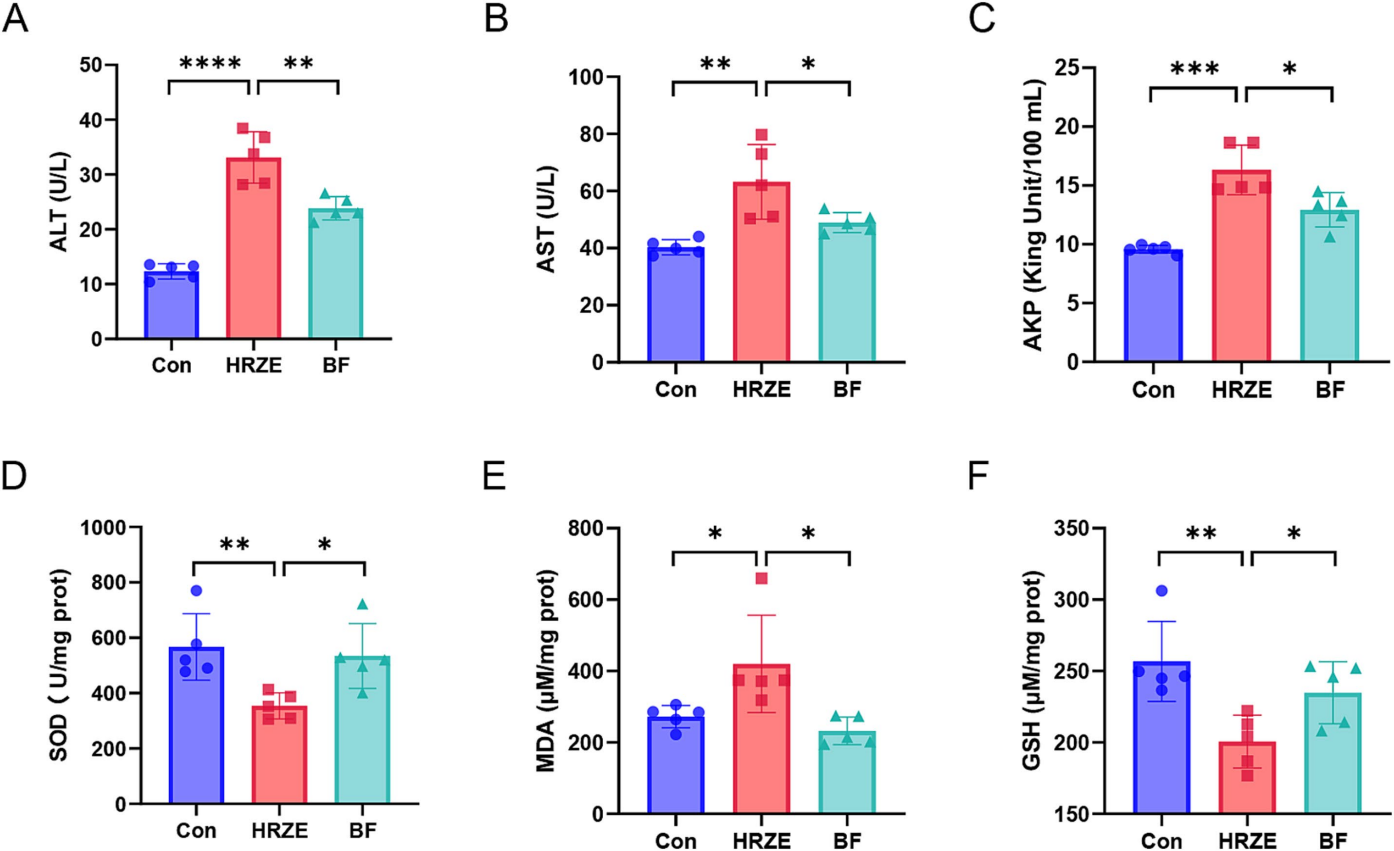
*Bacteroides fragilis* 839 (BF839) alleviates liver injury induced by isoniazid, rifampicin, pyrazinamide, and ethambutol (HRZE). **(A)** Serum levels of alanine aminotransferase (ALT), **(B)** aspartate aminotransferase (AST), and **(C)** alkaline phosphatase (AKP); **(D)** Liver tissue homogenate levels of superoxide dismutase (SOD), **(E)** malondialdehyde (MDA), and **(F)** glutathione (GSH) in each group. A total of ten mice were used per group for analysis. To simplify the representation, data from five randomly selected mice per group are shown. Values are presented as the means ± standard error of the mean (SEM). *p < 0.05, **p < 0.01, ***p < 0.001, and ****p < 0.0001. Con, control group; HRZE, isoniazid + rifampicin + pyrazinamide + ethambutol model group; BF, HRZE + BF839 group.

As shown in Figure 1, the serum levels of ALT, AST, AKP, and the liver index were significantly higher (*p* < 0.05) in the HRZE group than those of the control group, indicating liver damage induced by HRZE treatment (Figures 1A–C). In addition, BF839 treatment led to a significant response in the levels of ALT, AST, and AKP (Figures 1A–C). Furthermore, compared to the control group, the HRZE group’s MDA level rose while the liver tissue’s SOD and GSH levels dramatically dropped (*p* < 0.05) (Figures 1D–F). In contrast, these indices were significantly reversed in the BF839-treated mice (Figures 1D–F), indicating that BF839 may provide protection against liver damage induced by HRZE.

**Figure 2:**
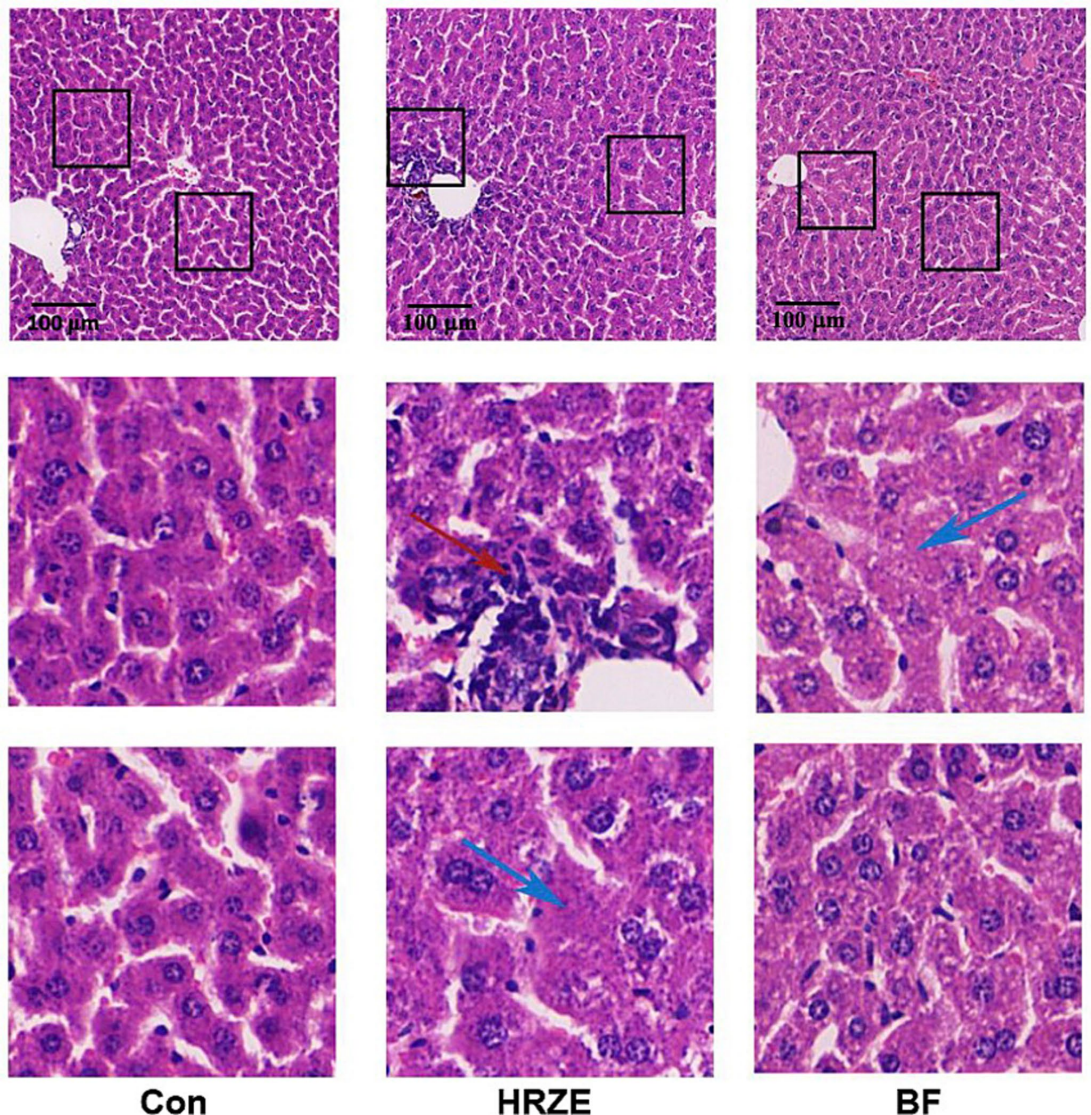
BF839 mitigates liver pathological alterations. Representative hematoxylin and eosin (H&E)-stained images of liver parenchyma (scale bar = 100 μm). Blue arrows indicate lipid vacuoles; the red arrow represents inflammatory cell infiltration. Con, control group; HRZE, isoniazid + rifampicin + pyrazinamide + ethambutol model group; BF, HRZE + BF839 group.

To visually assess the effect of BF839 on the liver, mouse liver sections were subjected to H&E staining (Figure 2). In the control group, the hepatic lobule structure and most hepatocytes appeared normal, with only mild edema observed in a few hepatocytes. However, the HRZE group showed varying degrees of hepatocyte edema and mild steatosis. Additionally, necrosis was observed in some hepatocytes, along with localized infiltration of inflammatory cells. In the HRZE+BF839 group, hepatocyte edema and steatosis were markedly reduced, and inflammatory cell infiltration was less prominent compared to the HRZE group. These findings indicate that BF839 has a protective effect against HRZE-induced liver injury and inflammation.

### 3.2 BF839 mitigates the negative effects of HRZE on the intestinal barrier in mice

**Figure 3:**
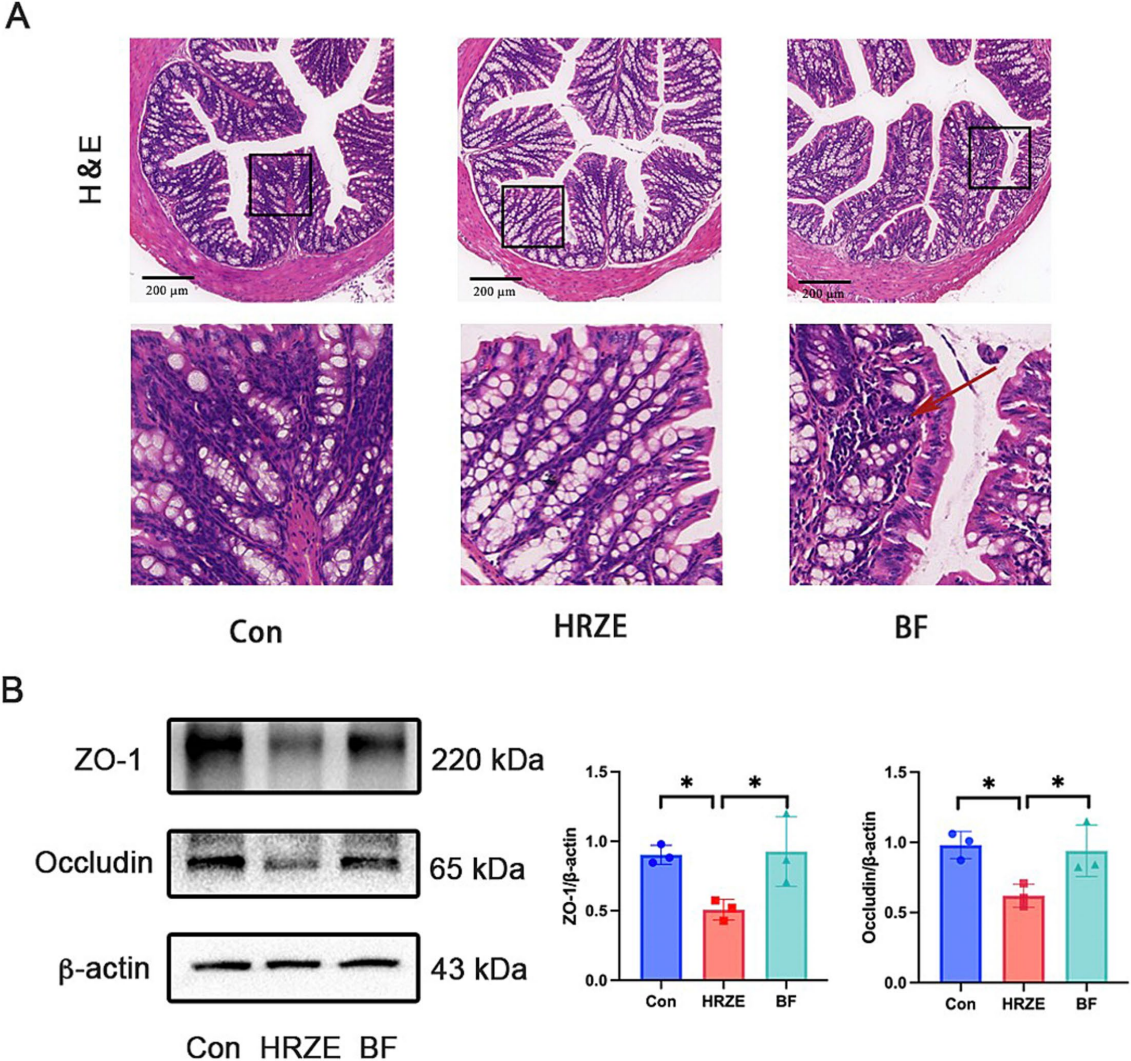
BF839 improves HRZE-induced intestinal barrier function. **(A)** Representative H&E-stained images of colon in each group (scale bar = 200 μm). Arrow represents inflammatory cell infiltration. **(B)** The protein levels of zona occludens (ZO)-1 and occludin in colon tissues analyzed by Western blot assays. Data are presented as means ± SEM, N = three per group. *p < 0.05. Con, control group; HRZE, isoniazid + rifampicin + pyrazinamide + ethambutol model group; BF, HRZE + BF839 group.

HRZE has a negative effect on the intestinal barrier, and the protective effects of BF839 on intestinal damage require further investigation. To assess this, H&E staining was used to detect the pathological changes (Figure 3A). In the HRZE group, although all layers were normal, the number of goblet cells in the mucosal layer was notably increased, whereas the numbers of absorptive colonocytes and intestinal stem cells were reduced compared to the control group. This suggested that HRZE induces goblet cell hyperproliferation and promotes differentiation of intestinal stem cell into goblet cells, likely to support intestinal regeneration and epithelial functions (Figure 3A). However, BF839 treatment mitigated the increased goblet cells (Figure 3A).

To further evaluate intestinal barrier function, the expression levels of representative tight junction proteins were examined in all groups (Figure 3B). In the HRZE group, the proteins levels of ZO-1 and occludin were significantly down-regulated (*p* < 0.05) compared to the control group. BF839 treatment notably restored the protein levels of ZO-1 and occludin (*p* < 0.05) in the HRZE group. These results indicate that BF839 alleviates HRZE-induced intestinal barrier disruption in mice.

### 3.3 BF839 modulated the diversity of gut microbiota in HRZE-treated mice

**Figure 4:**
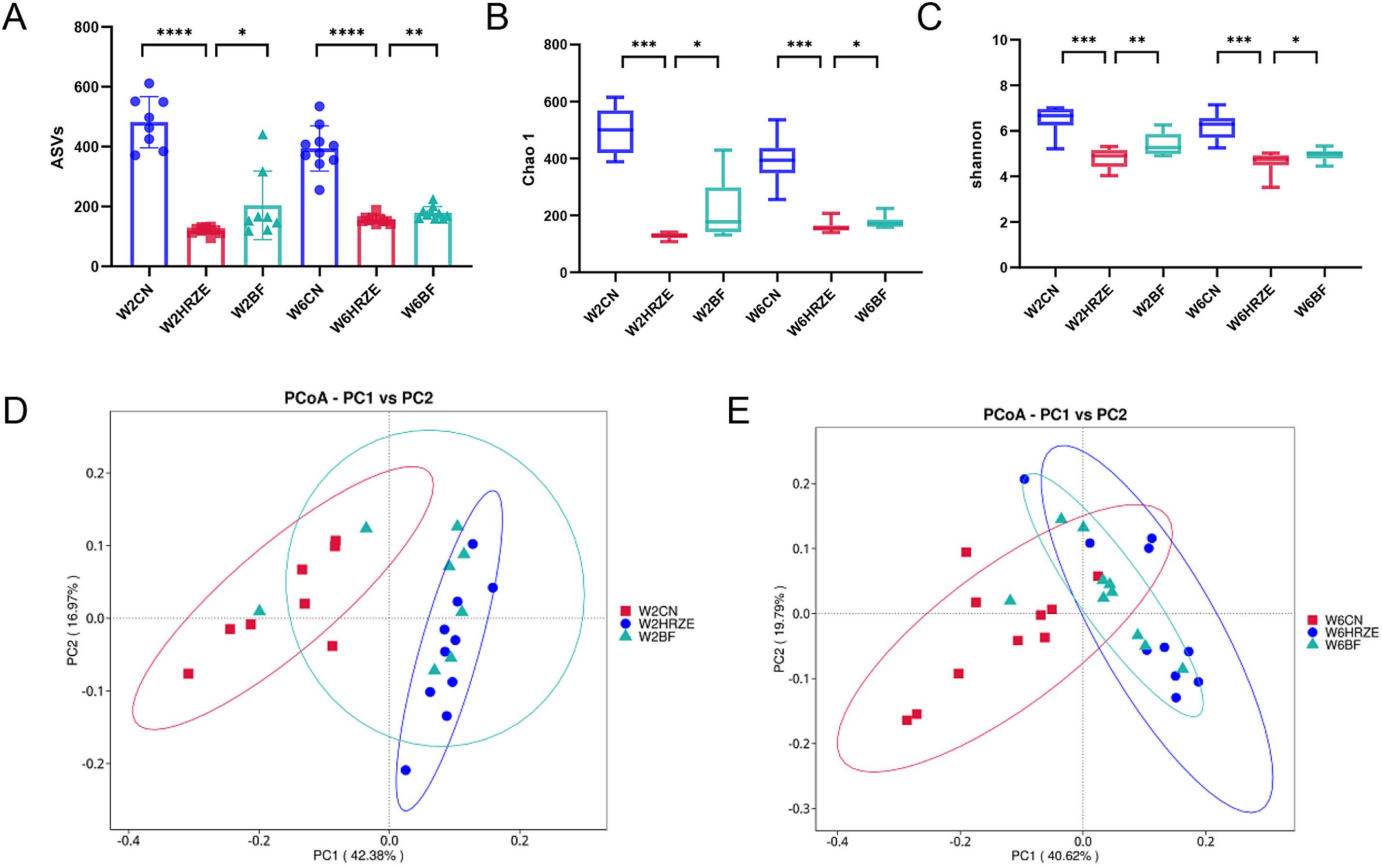
BF839 restores HRZE-induced reductions in alpha and beta diversity. Alpha diversity indicators: **(A)** Amplicon Sequence Variants (ASVs) counts, **(B)** Chao1 index, and **(C)** Shannon index. Beta diversity: **(D)** Principal Coordinates Analysis (PCoA) based on unweighted UniFrac distances at the ASV level for **(D)** week two and **(E)** week six. Data are presented as means ± SEM, N = eight per group. *p < 0.05, **p < 0.01, ***p < 0.001, and ****p < 0.0001. Con, control group; HRZE, isoniazid + rifampicin + pyrazinamide + ethambutol model group; BF, HRZE + BF839 group.

As an exogenous probiotic, BF839 may influence the composition of the intestinal microbiota. To verify this hypothesis, the fecal samples from mice at week two and week six were collected for 16S rRNA sequencing to analyze changes in microbiota composition. The relative abundance of bacteria was presented in Supplementary Table S1. The number of ASVs, an indicator of microbial richness, was significantly reduced in the HRZE group compared to the control group at both weeks two and six (*p* < 0.05, one-way ANOVA). However, the application of BF839 partially restored the ASV count, mitigating the effect of HRZE treatment (Figure 4A). Alpha diversity was assessed using the Chao1 and Shannon indices, which reflect microbial richness and diversity, respectively. At both weeks two and six, the index of the samples in the HRZE group was significantly lower compared to the control group (*p* < 0.05). Conversely, the indices in the BF group were significantly higher than those in the HRZE group (*p* < 0.05), indicating that BF839 improved microbial diversity disrupted by HRZE treatment (Figures 4B,C). Beta diversity was analyzed using unweighted UniFrac principal coordinates analysis (PCoA) to evaluate differences in microbial community composition between groups. At week two, the PCoA plot showed that the microbial community of HRZE was distinctly separated from those in the control group (*p* < 0.05, PERMANOVA). The HRZE+BF839 group exhibited partially overlapping clusters with both the control and HRZE groups, suggesting that BF839 modulated the microbiota toward a profile closer to the control. At week six, the microbiota profiles of the HRZE and HRZE+BF839 groups were slightly separated from that of the control group (*p* < 0.05, PERMANOVA), indicating a persistent, albeit attenuated, effect of BF839 on the gut microbiota (Figures 4D,E). These results demonstrate that BF839 can ameliorate HRZE-induced disruptions in gut microbiota diversity and composition by improving both microbial richness and community structure.

### 3.4 BF839 modulated microbial taxonomic profiles in HRZE-treated mice

**Figure 5:**
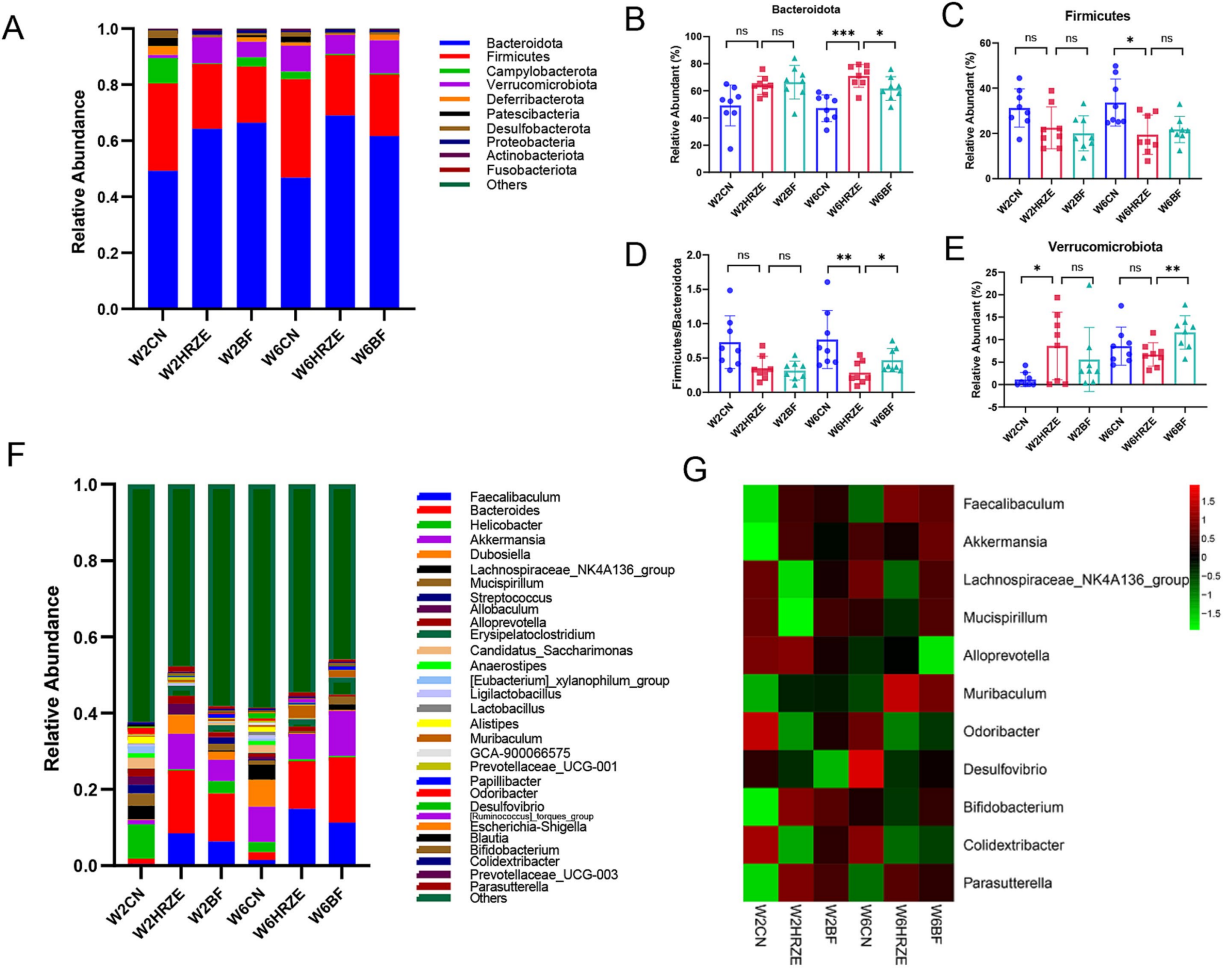
BF839 modulates HRZE-induced gut microbiota composition. **(A)** Relative abundance of microbial taxa at the phylum level, determined by 16S rRNA analysis of fecal samples. Relative abundances of gut microbial taxa at the phylum level: **(B)** Bacteroidetes; **(C)** Firmicutes; **(D)** Firmicutes to Bacteroidetes (F/B) ratio; **(E)** Verrucomicrobiota. **(F)** Genus-level species composition; **(G)** Relative abundances of gut microbial taxa at the genus level. Data are presented as means ± SEM, N = eight per group. *p < 0.05, **p < 0.01, ***p < 0.001, and ****p < 0.0001. Con, control group; HRZE, isoniazid + rifampicin + pyrazinamide + ethambutol model group; BF, HRZE + BF839 group.

As shown in Figure 5, change in microbiome profiles were analyzed from the phylum to genus levels. The ten most abundant phyla and thirty most abundant genera revealed significant differences in gut microbiota compositions among the groups (*p* < 0.05, one-way ANOVA).

At the phylum level, Bacteroidota accounted for the largest proportion across all groups (Figure 5A). The relative abundance of Bacteroidota were increased significantly in the HRZE treatment at week two and week six compared to the Con group (*p* < 0.05; Figure 5B). In addition, Firmicutes enrichment was significantly reduced at week six in the HRZE group (*p* < 0.05; Figure 5C). Following BF839 supplementation, the relative abundance of Bacteroidota was decreased significantly at week six compared to the HRZE group (*p* < 0.05; Figure 5B). In addition, the ratio of Firmicutes to Bacteroidota (F/B) in the HRZE group was significantly lower than that in the control group (*p* < 0.05), while BF839 administration significantly increased the F/B ratio compared to the HRZE group at week six (*p* < 0.05; Figure 5D). BF839 treatment also significantly increased the relative abundance of Verrucomicrobiota in the HRZE group at week six (*p* < 0.05; Figure 5E).

At the genus level, significant alterations were observed in the relative abundance of eleven key genera, including Faecalibaculum, Akkermansia, Lachnospiraceae_NK4A136_group, Mucispirillum, Alloprevotella, Muribaculum, Odoribacter, Desulfovibrio, Bifidobacterium, Colidextribacter, and Parasutterella (Figures 5F,G). Specifically, Faecalibaculum, Muribaculum, and Parasutterella showed significantly increased relative abundance in the HRZE group compared to the control group (*p* < 0.05), whereas the relative abundance of Akkermansia, Lachnospiraceae_NK4A136_group, Mucispirillum, Alloprevotella, Odoribacter, Desulfovibrio, Bifidobacterium, and Colidextribacter decreased significantly (*p* < 0.05). However, BF839 treatment significantly improved the abundance of Akkermansia, Lachnospiraceae_NK4A136_group, Mucispirillum, Alloprevotella, Odoribacter, Desulfovibrio, Bifidobacterium, and Colidextribacter and significantly reduced the abundance of Faecalibaculum, Muribaculum, and Parasutterella (*p* < 0.05). These findings suggest that BF839 modulates HRZE-induced dysbiosis by restoring the gut microbiota toward a healthier composition.

### 3.5 BF839 improved the liver inflammatory response mediated by HRZE via the LPS/TLR4/NF-kB/MyD88 signaling pathway

**Figure 6:**
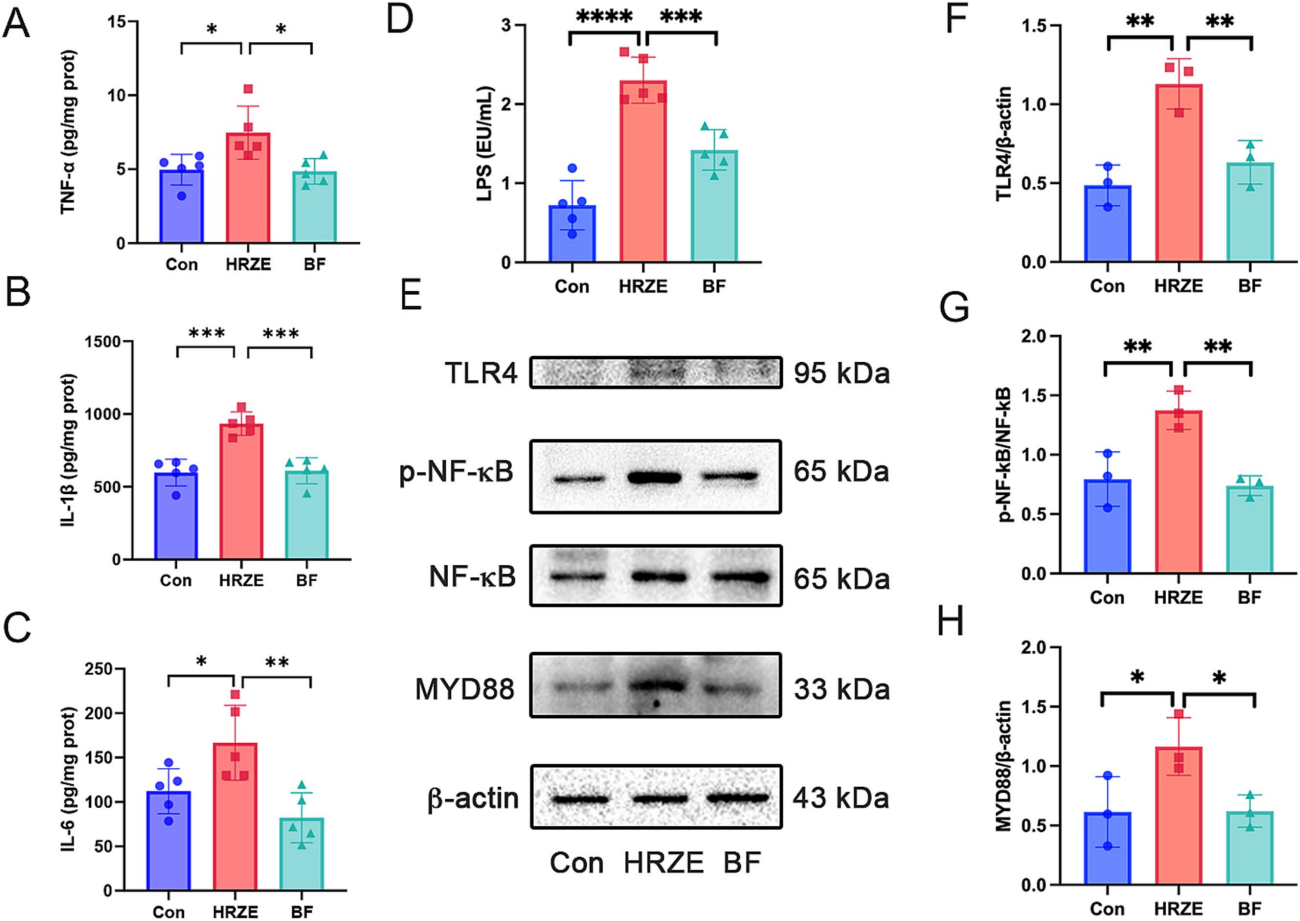
BF839 inhibits the inflammatory response and the lipopolysaccharide (LPS)/toll-like receptor 4 (TLR4)/nuclear factor kappa B (NF-kB)/*myeloid differentiation factor* 88 (MyD88) signaling pathway induced by HRZE. **(A)** Tumor necrosis factor-alpha (TNF-α) levels; **(B)** Interleukin (IL)-6 levels; **(C)** IL-1β levels; **(D)** Serum LPS levels; **(E)** Protein expression of TLR4, NF-κB, and MyD88 measured by Western blot; **(F)** Quantitative analysis of TLR4 protein expression; **(G)** Quantitative analyses of NF-κB and p- NF-κB protein expression; **(H)** Quantitative analysis of MyD88 protein expression. Data are presented as the means ± SEM, N = five per group for LPS, N = three per group for protein expression. *p < 0.05, **p < 0.01, ***p < 0.001, and ****p < 0.0001. Con, control group; HRZE, isoniazid + rifampicin + pyrazinamide + ethambutol model group; BF, HRZE + BF839 group.

To evaluate the degree of inflammation, the levels of proinflammatory cytokines TNF-α, IL-1β, and IL-6 were measured in liver tissues from each group of mice. The levels of TNF-α, IL-1β, and IL-6 in HRZE group were significantly elevated (*p* < 0.05) compared to the control group (Figures 6A–C). However, BF839 treatment significantly reduced (*p* < 0.05) the HRZE-induced increases in TNF-α, IL-1β, and IL-6 levels. These results suggest that BF839 alleviates HRZE-induced pro-inflammatory response in liver tissue of mice.

To explore whether HRZE-induced pro-inflammatory response was mediated by the LPS/TLR4/NF-κB signaling pathway, we measured the LPS level in mouse serum and the protein levels associated with the NF-κB signaling pathway. HRZE treatment increased serum LPS levels, while BF839 treatment significantly decreased (*p* < 0.05) LPS secretion (Figure 6D). Additionally, HRZE treatment enhanced the phosphorylation of NF-κB and up-regulated the expression levels of TLR4 and MyD88. Conversely, BF839 treatment mitigated these effects (Figures 6E–H). These results indicate that BF839 may attenuate HRZE-induced liver injury by inhibiting LPS/TLR4/NF-κB/MyD88 signaling pathway.

### 3.6 The correlation analysis of gut microbiota and indexes of serum liver function, oxidative stress and inflammation

**Figure 7:**
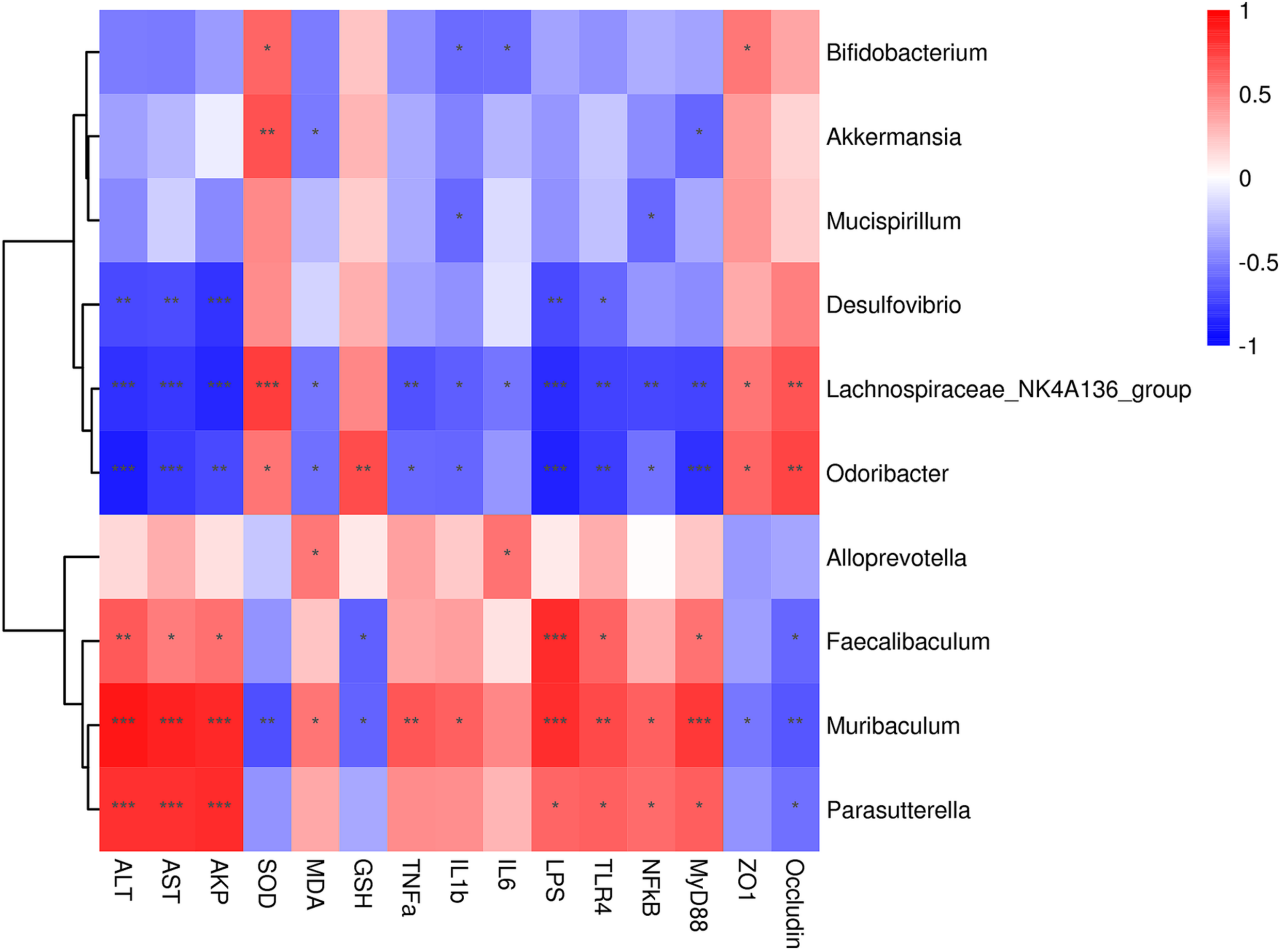
Spearman correlation analysis of intestinal microbes at the genus level and serum liver function, oxidative stress and inflammation indices. Correlation analysis between gut microbiota and serum liver function, oxidative stress and inflammation indices across all the groups was performed using Spearman’s correlation analysis. N = eight in per group. The correlation coefficient threshold is set to 0.5, *p < 0.05, **p < 0.01; ***p < 0.001.

The correlation analysis between the gut microbiota biomarkers at genus level and the indices of serum liver function, oxidative stress, inflammation is presented in Figure 7. Desulfovibrio, Lachnospiraceae_NK4A136_group and Odoribacter exhibited a negative connection with ALT, AST and AKP levels, while showing positive associations with ZO-1 and Occludin. Conversely, Faecalibaculum, Muribaculum and Parasutterella were positively correlated with serum liver function induces and negatively correlatied with tight junction proteins. Furthermore, Bifidobacterium, Akkermansia, Lachnospiraceae_NK4A136_group, Odoribacter, Alloprevotella, Faecalibaculum, and Muribaculum were associated with oxidative stress and inflammation indices. Specifically, Bifidobacterium, Akkermansia, Lachnospiraceae_NK4A136_group and Odoribacter demonstrated negative correlations with MDA, TNF-α, IL-1β, and IL-6, while showing positive correlations with SOD and GSH. However, Alloprevotella, Faecalibaculum, and Muribaculum exhibited positive correlations with MDA, TNF-α, IL-1β, and IL-6, and negative correlations with SOD and GSH. Regarding, the LPS/TLR4/NF-κB pathway, Desulfovibrio, Lachnospiraceae_NK4A136_group, and Odoribacter showed negative correlations, whereas Faecalibaculum, Muribaculum and Parasutterella demonstrated positive correlations.

## 4 Discussion

The data presented above confirm that BF839 could protect against HRZE-induced liver injury in mice by correcting gut microbiota imbalance and improving intestinal barrier function. ATB-DILI adversely affects the treatment and prognosis of TB. The gut–liver axis, which represents the interaction between the gut microbiota and the liver, plays a critical role in hepatic drug metabolism and contributes to the development of ATB-DILI. Previous studies have suggested that various probiotics, including *Lactobacillus casei* and *Lactobacillus rhamnosus*, can ameliorate ATB-DILI by restoring gut dysbiosis (11, 20, 21). In this study, an ATB-DILI model was established by exposing mice to HRZE. The results demonstrated that BF839 alleviated HRZE-induced liver injury, as evidenced by reduced liver enzymes (ALT, AST and AKP) levels, improved oxidative stress markers (increased SOD and GSH levels and reduced MDA levels), regulated gut microbiota composition, repaired the intestinal barrier, attenuated inflammation response by modulating LPS release into the bloodstream and the TLR4/NF-κB/MyD88 pathway in liver.

Serum ALT and AST levels are critical and sensitive indicators of liver function, including DILI (3). In this study, HRZE administration significantly increased ALT, AST, and AKP levels, accompanied by liver pathological changes. Supplementation with BF839 significantly alleviated HRZE-induced liver injury in mice. Oxidative stress plays a pivotal role in ATB-DILI (22, 23). The antioxidant enzyme systems, including SOD, MDA, and GSH, are commonly used to evaluate oxidative stress. SOD activity indirectly reflects the body’s ability to scavenge oxygen free radicals, while MDA level can reflect lipid peroxidation levels. In addition, GSH, an essential antioxidant and free radical scavenger, enhances immunity and reduces inflammation. In this study, MDA levels were significantly elevated in the liver tissues of HRZE-treated mice, while SOD and GSH levels were significantly reduced. However, BF839 treatment decreased MDA levels and increased SOD and GSH levels, indicating that BF839 mitigates HRZE-induced liver injury.

The gut-liver axis plays a crucial role in the onset of the development of liver damage and various hepatic disorders (24, 25). It represents a bidirectional interaction between the gastrointestinal tract, its microbiota, and the liver, primarily mediated through the portal circulation. The gut mucosal barrier is mainly composed of gut microbial balance and intestinal epithelial cells (24). Recent studies have highlighted the relationship between gut microbiota, gut barrier function, and ATB-DILI (11, 20, 21). The crypt-villus architecture and the rapid turnover of the intestinal epithelium, which are dependent on intestinal stem cells located in the crypts, are essential for maintaining the gut’s absorptive and barrier functions (26, 27). Intestinal stem cells differentiate into transit-amplifying cells, which subsequently develop into secretory or absorptive cells and migrate along the villi (28, 29). In response to injury, intestinal stem cells are activated to promote epithelial function and regeneration. In this study, HRZE exposure induced goblet cell hyperproliferation and promoted intestinal stem cells differentiation into goblet cells, indicating increased epithelial damage. In addition, inflammation was observed in the colonic mucosa of HRZE-treated mice. However, BF839 treatment reduced goblet cell proliferation, increased intestinal stem cell populations, and reduced inflammatory cell infiltration. Furthermore, BF839 significantly up-regulated the expression of Tight junction proteins, such as ZO-1 and occludin, compared to HRZE-treated mice, suggesting that BF839 may improve ATB-DILI-induced intestinal barrier dysfunction.

Some investigations reported that anti-TB medicines reduced the diversity of the gut microbiome (11, 20, 21, 30). In this work, we observed similar findings, as HRZE treatment decreased the number of ASVs, reduced alpha diversity indices, and altered the composition of the intestinal microbiota in mice. However, BF839 supplementation restored the diversity and structure of the gut microbiota and contributed to maintaining microbial balance. Firmicutes and Bacteroides are the two dominant bacterial phyla that make up the majority of the human gut microbiota. Firmicutes being the most abundant phylum in the colon. Bacteroides, which include conditional pathogenic bacteria, exhibit pro-inflammatory properties (31). In mice exposed to HRZE, the abundance of Bacteroides increased, while Firmicutes and Verrucomicrobiota levels declined. BF839 supplementation improved the relative abundance of Firmicutes and Verrucomicrobiota and increased Firmicutes/Bacteroidetes ratio. At the genus level, HRZE treatment induced the higher abundance of Muribaculum, a member of the Bacteroidetes family, which acts as a pathobiont in dysbiotic gut microbiota (32). Furthermore, HRZE-treated mice exhibited lower levels of potential probiotics, including Akkermansia, Lachnospiraceae_NK4A136_group, and Bifidobacterium. BF839 supplemention restored the relative abundances of these beneficial genera. Notably, Akkermansia is known to promote intestinal stem cell proliferation and differentiation, enhance thicken mucus, and repair intestinal mucosa injury (33). The findings suggested that BF839 may facilitate gut epithelial regeneration through potential probiotic-mediated mechanisms.

LPS, produced by Gram-negative bacteria (34), is closely associated with the value of Firmicutes/Bacteroidetes. Disruption of gut microbial balance increases intestinal permeability, allowing more LPS to enter the bloodstream (35). In this study, HRZE-treated mice exhibited significantly elevated blood LPS levels, whereas BF839 supplementation effectively reduced these levels. LPS activates TLR4, triggering the release of key pro-inflammatory cytokines necessary for initiating robust immune responses (34). The TLR4/NF-κB pathway plays a critical role in initiating a pro-inflammatory cascade when LPS enters the bloodstream, particularly in ATB-DILI-induced liver injury (11, 21). This process results in the TNF-α and IL-6 production (36). Our findings demonstrated that HRZE administration increased TNF-α, IL-6, and IL-1β levels and activated the TLR4/NF-κB/MyD88 pathway in liver tissue, which was associated with elevated LPS levels. Conversely, BF839 treatment suppressed the expression of TLR4, phosphorylated NF-κB, MyD88, as well as HRZE-induced inflammatory factors. These results suggest that BF839 may mitigate ATB-DILI by inhibiting LPS/TLR4/NF-κB/MyD88 signaling pathway.

## 5 Conclusion

In conclusion, our study demonstrated that probiotics exert protective effects against ATB-DILI. BF839 mitigated HRZE-induced liver injury by restoring gut microbiota, enhancing intestinal barrier integrity, and decreasing inflammation through the LPS/TLR4/NF-κB signaling pathway. These results provide theoretical support for the potential use of BF839 as a potential probiotic therapy option for ATB-DILI patients. Future studies will focus on evaluating the protective effects of BF839 on ATB-DILI in human subjects.

## Data Availability

The datasets presented in this study can be found in online repositories. The names of the repository/repositories and accession number(s) can be found in the article/Supplementary material.

## Footnotes

^1^ https://magic.novogene.com/customer/main#/homeNew

^2^ www.biocloud.net

## Glossary

ATB-DILI: anti-tuberculosis drug-induced liver injury
anti-TB: anti-tuberculosis
ASVs: Amplicon Sequence Variants
AST: aspartate aminotransferase
ALT: alanine aminotransferase
AKP: alkaline phosphatase
***B. fragilis***: *Bacteroides fragilis*
BF839: *Bacteroides fragilis* 839
DILI: drug-induced liver injury
ELISA: enzyme-linked Immunosorbent Assay
GSH: glutathione
H&E: hematoxylin and eosin
HRZE: isoniazid, rifampicin, pyrazinamide and ethambutol
IL: interleukin
LPS: lipopolysaccharide
MDA: malondialdehyde
MyD88: *myeloid differentiation factor* 88
NF-κB: nuclear factor kappa B
OTU: Operational taxonomic unit
SEM: means ± standard error of the mean
ANOVA: one-way analysis of variance
PMSF: phenylmethanesulfonyl fluoride
SOD: superoxide Dismutase
SEM: standard error of the mean
SPF: specific pathogen-free
TLR4: Toll-like receptor 4
TB: Tuberculosis
TNF-α: tumor necrosis factor-alpha
ZO-1: zona occludens-1
